# Corticosteroid eye drop instillation aggravates the development of *Acanthamoeba* keratitis in rabbit corneas inoculated with *Acanthamoeba* and bacteria

**DOI:** 10.1038/s41598-019-49128-7

**Published:** 2019-09-06

**Authors:** Hayate Nakagawa, Naohito Koike, Tomoko Ehara, Takaaki Hattori, Akitomo Narimatsu, Shigeto Kumakura, Hiroshi Goto

**Affiliations:** 1 Department of Ophthalmology, Tokyo Medical University, Shinjuku City, Japan; 2 Department of Microbiology, Tokyo Medical University, Shinjuku City, Japan

**Keywords:** Corneal diseases, Parasitic infection

## Abstract

The role of topical corticosteroids in management of *Acanthamoeba* keratitis (AK) remains controversial. Using a rabbit AK model, we investigated whether corticosteroid use is a risk factor of AK. *Acanthamoeba* (1 × 10^5^/ml) was incubated with two densities of *P*. *aeruginosa* (PA; high-PA: 1 × 10^8^/ml, low-PA: 3 × 10^5^/ml) before corneal inoculation. Rabbit corneas were inoculated with *Acanthamoeba* alone or *Acanthamoeba* plus PA and administered levofloxacin and betamethasone sodium phosphate (BSP) eye drops for 5 or 7 days. Infected rabbit eyes were evaluated for clinical score and *Acanthamoeba* by histological examination. *Acanthamoeba* alone and BSP treatment did not produce keratitis. Corneas inoculated with *Acanthamoeba* plus low-PA treated immediately with levofloxacin and BSP remained clear with few infiltrates. Corneas inoculated with *Acanthamoeba* plus low-PA treated with levofloxacin immediately and BSP 12 h later developed severe keratitis. Corneas inoculated with *Acanthamoeba* plus high-PA treated immediately with levofloxacin and BSP also developed severe keratitis. Acanthamoebae were detected by PAS staining in corneas inoculated with *Acanthamoeba* plus high-PA treated with levofloxacin and BSP. Topical corticosteroids have the potential to aggravate AK when cornea is infected by *Acanthamoeba* with a critical number of bacteria or when corticosteroids are given after infection has established by *Acanthamoeba* with small number of bacteria.

## Introduction

*Acanthamoeba* keratitis (AK) is a rare but severe corneal infection. This painful, sight-threatening, and difficult-to-treat corneal infection is caused by an opportunistic protist belonging to the genus *Acanthamoeba*^1–3^. The treatment of AK has not been satisfactory and requires improvement^4^. Topical corticosteroids are frequently needed to control pain and inflammation in AK^5^. However, the role of topical corticosteroids in the management of AK remains controversial^6–9^. Robaei *et al*.^8^ reported that corticosteroid use before diagnosis of AK is highly predictive of poorer visual outcome. McClellan *et al*.^9^ also found that exposure of *Acanthamoeba* trophozoites and cysts to dexamethasone increases the pathogenicity of the organisms. From these reports, corticosteroid use may be a risk factor for the development or progression of AK. We therefore investigated whether corticosteroid use is a risk factor of AK *in vivo*; and if it is a risk factor, how corticosteroids affect the pathology of AK.

Recently, much interest has been focused on the bacterial symbionts extracted from *Acanthamoeba* strains isolated from patients with AK^10–13^. We have investigated the relationship between bacteria and *Acanthamoeba* in the development of keratitis using a rabbit AK model to reveal the pathogenic mechanisms of AK^14,15^. Our previous investigations showed that the presence of bacteria may be indispensable and a critical number of bacteria with adequate time for *Acanthamoeba*‒bacteria interaction may be required for the development of AK. Our previous experimental AK animal model using a clinical isolate of *Acanthamoeba* strain and *E*. *coli* showed the development of severe keratitis^14^. Moreover, the *Acanthamoeba* strain ATCC 50492 and *P*. *aeruginosa* strain PAO-1 caused keratitis that peaked at days 1–2 and thereafter attenuated gradually^15^. It remains unclear why the development of AK weakened. We speculated that *P*. *aeruginosa* is not capable of accelerating the development of AK, and that the bacterial species and the interaction with *Acanthamoeba* may be important for the development of AK.

In the present study, we investigated the effect of betamethasone sodium phosphate (BSP) eye drop in a rabbit AK model produced by inoculation of *Acanthamoeba castellanii* ATCC 50492 and *P*. *aeruginosa* strain PAO-1. We also investigated the interaction between the two organisms in the presence of corticosteroids.

## Results

### Inoculation of *acanthamoeba* alone and treatment with BSP eye drops did not induce keratitis

**Figure 1:**
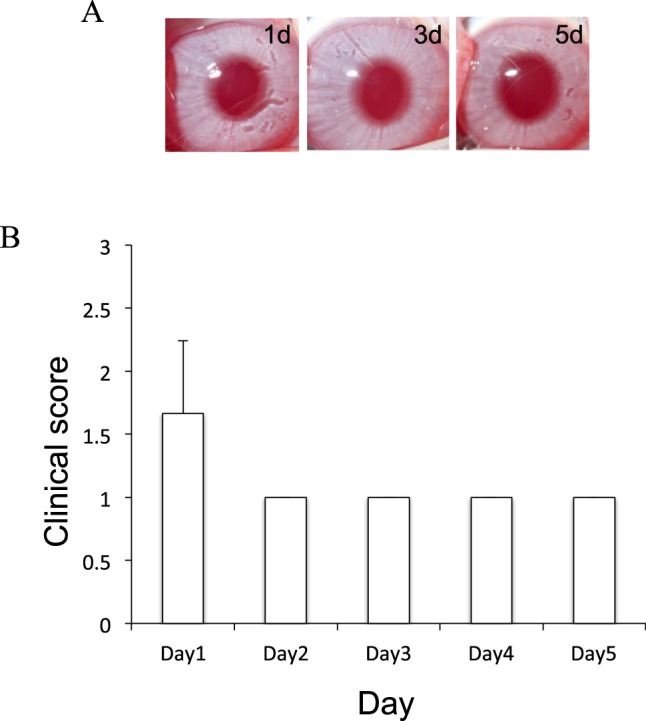
Clinical results of rabbit corneas inoculated with *Acanthamoeba* alone in which BSP eye drops three times daily was started immediately after inoculation. (**A**) Representative rabbit corneal photographs on days 1–5 after inoculation with *Acanthamoeba* alone and administration of BSP eye drops 3 times a day. The corneas remain clear. (**B**) Rabbit corneas inoculated with *Acanthamoeba* alone and treated with BSP eye drops shows few infiltrates and mild inflammation in the cornea.

We examined whether inoculation of the *Acanthamoeba* strain (ATCC 50492) alone followed by treatment with BSP eye drops induces keratitis. After rabbit corneas were inoculated with *Acanthamoeba*, BSP eye drops 3 times a day were started immediately. The corneas were examined microscopically after inoculation on day 0 until day 5. Rabbit corneas inoculated with *Acanthamoeba* and treated with BSP eye drops showed few infiltrates on day 1 post-infection (Fig. 1A,B). The corneas remained clear during the experimental period. Based on our definition of clinical end point, the rabbits inoculated with *Acanthamoeba* were euthanized on day 5.

### Antibiotic and corticosteroid treatment of corneas inoculated with *acanthamoeba* plus low- density *P. aeruginosa*

**Figure 2:**
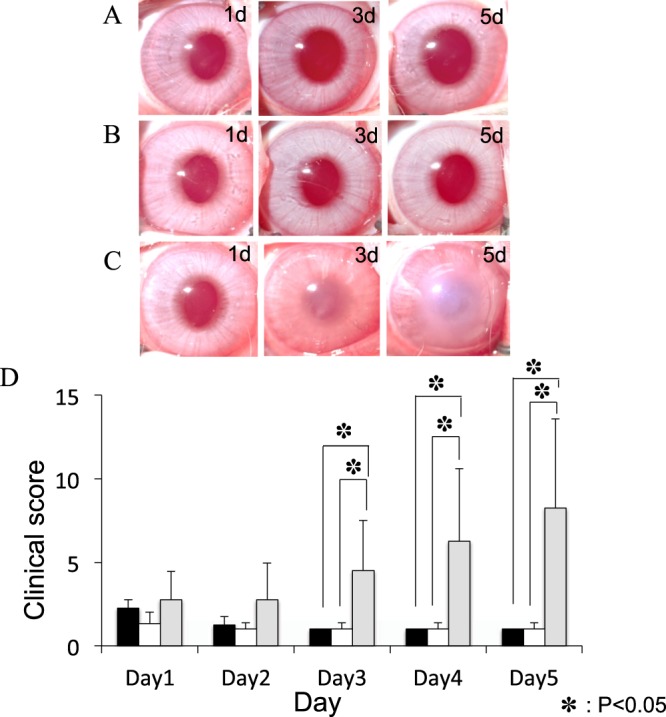
Clinical results of treatment with LVFX eye drop alone or with LVFX and BSP eye drops for keratitis produced by inoculating *Acanthamoeba* with a low density of *P. aeruginosa*. (**A**–**C**) Representative photographs (days 0–5) of keratitis in rabbit eyes inoculated with *Acanthamoeba* (1 × 10^5^/ml) plus low-density *P*. *aeruginosa* (3 × 10^5^/ml) followed immediately by treatment with 1.5% LVFX eye drops 3 times a day (**A**), or with 1.5% LVFX eye drops together with BSP eye drops 3 times a day (**B**), or immediately with 1.5% LVFX eye drops and 12 h later with BSP eye drops, both 3 times a day (**C**). (**D**) Clinical scores (days 1–5 post-inoculation) of keratitis in the above three groups. Closed bar denotes treatment immediately after inoculation with 1.5% LVFX eye drops; open bar denotes treatment immediately after inoculation with 1.5% LVFX + BSP eye drops; and gray bar denotes treatment immediately after inoculation with 1.5% LVFX followed by BSP eye drops 12 h later. Keratitis is significantly more severe in the group treated with BSP eye drops after a delay following inoculation compared to the other two groups. ^*^P < 0.05, n = 4 in each group.

In our previous study, corneas inoculated with a mixture of *Acanthamoeba* plus low-density *P*. *aeruginosa* and treated immediately with LVFX eye drops showed few infiltrates on day 1 post-infection and remained clear during the experiment. To investigate the effect of corticosteroids, we used a similar experimental infection model and compared the clinical scores of keratitis produced by *Acanthamoeba* plus *P*. *aeruginosa* treated with LVFX with or without corticosteroid eye drop. We treated rabbit corneas with 1.5% LVFX eye drops or 1.5% LVFX eye drops and BSP eye drops immediately after inoculation of *Acanthamoeba* plus low-density *P*. *aeruginosa*. The corneas in both groups showed few infiltrates on day 1 post-infection and remained clear during the experiment (Fig. 2A,B). There were no differences in clinical score between the two groups during the observation period (Fig. 2D).

We also investigated how corticosteroid affects the cornea in which *Acanthamoeba* infection has already established. Corneas inoculated with *Acanthamoeba* plus low-density *P*. *aeruginosa* were treated with 1.5% LVFX eye drops immediately followed by BSP eye drops 12 h after inoculation. These corneas showed few infiltrates on day 1 post-infection, but developed mild infiltrates at the center of the cornea with diffuse corneal edematous on day 3 post-infection, and progressed to severe keratitis on day 5 post-infection (Fig. 2C).

Keratitis was significantly more severe in the group treated with BSP eye drops after a lapse of 12 h following inoculation compared with the other two groups from day 3 to day 5 (Fig. 2D).

### Antibiotic or antibiotic and corticosteroid treatment of corneas inoculated with *acanthamoeba* plus high-density *P. aeruginosa*

**Figure 3:**
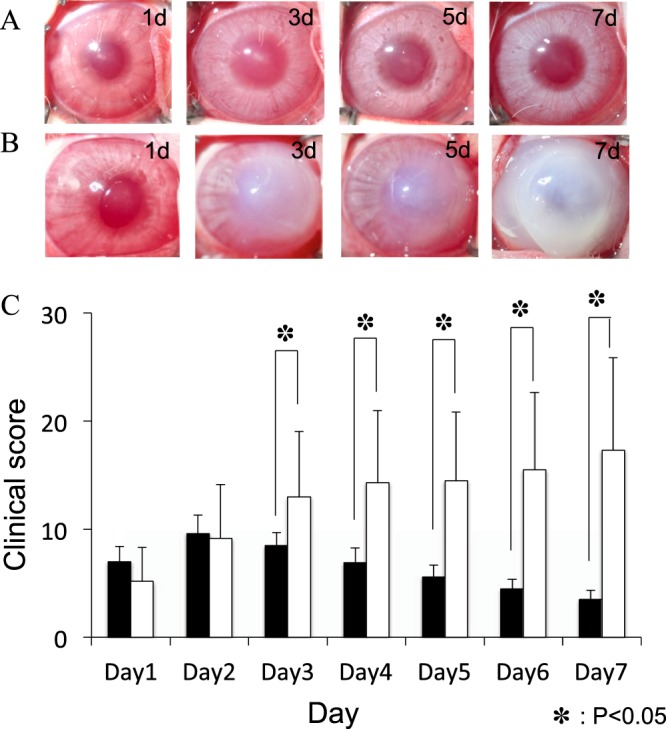
Clinical results of treatment with LVFX eye drop alone or with LVFX and BSP eye drops for keratitis produced by inoculating *Acanthamoeba* with a high density of *P. aeruginosa*. (**A**,**B**) Representative photographs of keratitis in rabbit eyes inoculated with *Acanthamoeba* (1 × 10^5^/ml) plus high-density *P*. *aeruginosa* (1 × 10^8^/ml) and treated immediately with 1.5% LVFX eye drops 3 times a day (**A**), or with 1.5% LVFX eye drops and BSP eye drops 3 times a day (**B**). (**C**) Clinical scores (days 1–5 post-inoculation) of keratitis in the above two groups. Closed bar denotes treatment immediately after inoculation with 1.5% LVFX eye drops; and open bar denotes treatment immediately after inoculation with 1.5% LVFX + BSP eye drops. Clinical scores are significantly higher in inoculated corneas treated with LVFX and BSP compared with LVFX alone. ^*^P < 0.05, n = 4 in each group.

In our previous study^15^, corneas inoculated with *Acanthamoeba* pre-incubated with a high density of *P*. *aeruginosa* and treated with LVFX developed severe keratitis that peaked at days 1–2 and thereafter attenuated gradually. We investigated the effect of corticosteroid using a similar keratitis model. Rabbit corneas inoculated with *Acanthamoeba* plus high-density *P*. *aeruginosa* treated immediately after inoculation with LVFX eye drops showed infiltration in the cornea and anterior chamber inflammation on days 1–2 post-infection. On day 3, infiltration in the central cornea was attenuated slightly and corneal edema was reduced. On day 5, corneal infiltration and corneal opacity were further reduced. (Fig. 3A). Consistent with previous report, the keratitis peaked at days 2 and thereafter attenuated gradually. On the other hand, rabbit corneas inoculated with *Acanthamoeba* plus high-density *P*. *aeruginosa* treated with LVFX eye drops and BSP eye drops developed severe keratitis (Fig. 3B). These eyes showed diffuse infiltration and edema in the cornea together with anterior chamber inflammation on days 1–3 post-infection. On day 5–7, diffuse infiltration in the cornea and corneal edema aggravated. On day 7, corneal infiltration further increased and corneal melting was observed. Significant differences in clinical score were observed between LVFX eye drops alone and LVFX with BSP eye drops from day 3 to day 7 (Fig. 3C).

### Pathological evidence of the presence of *acanthamoeba* in cornea tissue

**Figure 4:**
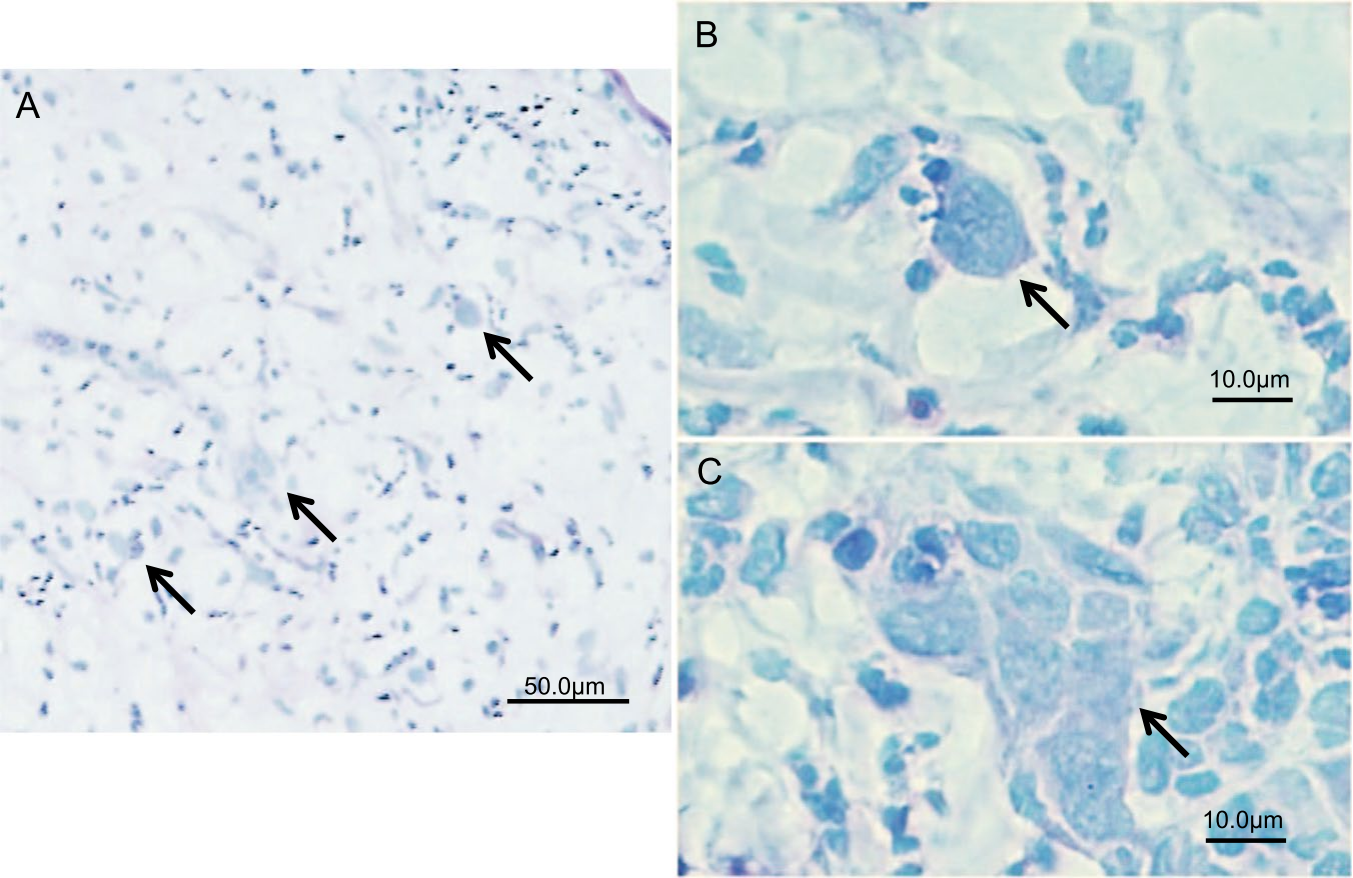
Histopathological evaluation by PAS staining of frozen sections of cornea inoculated with *Acanthamoeba* and a high density of *P. aeruginosa* followed by treatment with 1.5% LVFX and BSP eye drops. (**A**) Acanthamoebae (arrows) are observed in corneal stromal tissue under low magnification. (**B**,**C**) Under high magnification, structures resembling *Acanthamoeba* cysts are observed. In (**B**), *Acanthamoeba* cyst (arrow) is surrounded by phagocytes. In (**C**), aggregation of acanthamoebae (arrow) is observed.

We performed histopathological study to examine the existence of *Acanthamoeba* in rabbit corneas inoculated with *Acanthamoeba* plus high-density *P*. *aeruginosa* treated with LVFX and BSP eye drops. Initially, we used Fungiflora Y staining to detect *Acanthamoeba* cyst, but no *Acanthamoeba* cysts were detected in corneal tissue using this method, consistent with our previous finding^14^. Next, we performed PAS staining. Structures similar to *Acanthamoeba* cysts were detected in central corneal stroma (Fig. 4A). These structures were round, similar in shape to *Acanthamoeba* cysts, but without the specific double-wall structure of the *Acanthamoeba* cysts. The acanthamoebae were surrounded by phagocytes of the rabbit (Fig. 4B) and aggregated in central corneal stroma (Fig. 4C). We also investigated the histopathological findings of the corneas inoculated with *Acanthamoeba* plus low-density *P*. *aeruginosa* treated with levofloxacin immediately and with BSP 12 hours later. By PAS staining, we found no aggravation of acanthamoebae in corneal stroma, in contrast to the findings in corneas inoculated with *Acanthamoeba* plus high-density *P*. *aeruginosa* treated with BSP immediately. In histopathological examination, we found only mild corneal infiltration (Supplemental Fig. 1).

## Discussion

Our results showed that corneal inoculation of the *Acanthamoeba* strain (ATCC 50492) alone followed by treatment with BSP eye drops did not cause keratitis. Our previous report showed that a clinical isolate of *Acanthamoeba* strain alone without bacteria or a standard *Acanthamoeba* strain (ATCC 50492) alone did not induce keratitis in animal models^14,15^. Taken these results together, *Acanthamoeba* in the absence of symbionts may not be capable of producing keratitis regardless of the absence or presence of corticosteroids.

We evaluated the effects of topical corticosteroids on the development of AK in corneas inoculated with *Acanthamoeba* pre-incubated (2 h) with two densities of *P*. *aeruginosa*. Keratitis did not develop when corneas inoculated with *Acanthamoeba* plus low-density *P*. *aeruginosa* were treated immediately with 1.5% LVFX eye drops and BSP eye drops 3 times a day. The corneas remained almost clear during the experiment. On the other hand, severe keratitis developed when corneas inoculated with *Acanthamoeba* plus low-density *P*. *aeruginosa* were treated with 1.5% LVFX eye drops immediately after inoculation and additionally with BSP eye drops after a delay of 12 h. This result suggests that the pathological potential of *Acanthamoeba* is enhanced leading to the development of severe keratitis when topical corticosteroids are administered after *Acanthamoeba* infection has already established in the cornea. McClellan *et al*.^9^ established an AK animal model using Chinese hamsters and compared the severity of keratitis with and without systemic dexamethasone administration. Keratitis was more severe in animals treated with dexamethasone compared with untreated controls. The investigators administered dexamethasone to Chinese hamsters 24 h after corneal infection by *Acanthamoeba*. Despite the difference in experimental model, their study and ours both demonstrate that delayed treatment with corticosteroids increases the severity of keratitis in AK. We speculate that the cornea inoculated with *Acanthamoeba* plus low-density *P*. *aeruginosa* and treated immediately with LVFX develops mild keratitis, which may then regress because the pathological potential of *Acanthamoeba* is not adequate to accelerate corneal inflammation. On the other hand, topical corticosteroids administered 12 h after inoculation when *Acanthamoeba* infection has already established in the cornea may enhance the pathogenicity of *Acanthamoeba* to accelerate corneal inflammation.

Rabbit corneas inoculated with *Acanthamoeba* plus high-density *P*. *aeruginosa* and treated immediately with LVFX eye drops and BSP eye drops also developed severe keratitis resulting in corneal melting. In our previous study, the same animal model treated with LVFX but no BSP eye drops developed keratitis that peaked at day 2 and thereafter attenuated gradually^15^. The findings of our two studies suggest that *Acanthamoeba* pre-incubated with *P*. *aeruginosa* exceeding a critical number effectively establishes infection in the cornea promptly after corneal inoculation, and under such condition, exposure of *Acanthamoeba* to corticosteroids increases the pathogenicity of the organism accelerating the progression of corneal inflammation to a fulminant state.

In our previous study, we investigated the interaction of *Acanthamoeba* and bacteria in the development of AK, and showed the importance of the number of bacteria co-inoculated with *Acanthamoeba* in the development of AK^15^. In the present study, we found that the number of bacteria co-inoculated with *Acanthamoeba* also affected the effect of corticosteroids on the development of AK. When corneas are infected with *Acanthamoeba* and a low density of bacteria, the timing of administering topical corticosteroids may be important for the development of keratitis. We therefore recommend to administer antibiotic eye drops to reduce the number of bacteria as a measure to prevent severe AK in patients who are currently treated with topical corticosteroids.

In a study by McClellan *et al*.^9^, dexamethasone-treated trophozoites or cysts induced a significant cytopathic effect in corneal epithelial cells compared with untreated organisms. Their study indicates that corticosteroids may change the pathological potential of *Acanthamoeba*, which was observed in our *in vivo* study. Some studies suggest that corticosteroids inhibit encystment of *Acanthamoeba* trophozoites^7^, and that trophozoite proliferation is accelerated when trophozoites are treated with corticosteroids^9^. These studies imply that administration of corticosteroids may increase the ratio of trophozoites in AK. Our histopathological examination also showed that Fungiflora-Y staining detected no *Acanthamoeba* cysts, whereas PAS staining detected structures similar to *Acanthamoeba* cysts but lacked the specific double-wall structure of *Acanthamoeba* cysts. Our histopathological study thus suggest that corticosteroids may inhibit encystment of *Acanthamoeba* trophozoites. In a study by Garate *et al*.^16^, the pathogenic potential of *Acanthamoeba* correlates directly with the expression level of the parasite mannose-binding protein (MBP). *Acanthamoeba* cysts express markedly reduced levels of MBP compared with *Acanthamoeba* trophozoites. Extrapolating this finding to the results of our pathological examinations, we speculate that corticosteroids may induce transformation of the pathogenicity of *Acanthamoeba in vivo*, inhibiting encystment of *Acanthamoeba* trophozoites and upregulating the expression level of the MBP.

The present study has a limitation. The rabbit AK model and human AK differ in The mechanism of *Acanthamoeba* infection. In our model, *Acanthamoeba* is inoculated directly into corneal stromal tissues to produce severe keratitis. The merit of this animal model is the high efficiency of establishing keratitis and simple evaluation of inflammation. However, specific findings of human AK cannot be observed.

In conclusion, our study demonstrated that topical corticosteroids aggravated AK in rabbit corneas inoculated with *Acanthamoeba* pre-incubated with *P*. *aeruginosa*. Use of topical corticosteroids potentially leads to development of severe AK when administered immediately after the cornea is infected with *Acanthamoeba* containing a critical number of bacteria or when administered after a delay when *Acanthamoeba* containing a small number of bacteria has already established infection in the cornea. If topical corticosteroids have to be used in eyes affected by AK, administration of antibiotic eye drops to reduce the number of bacteria may be useful to prevent severe AK.

## Materials and Methods

### Cell cultures

All studies were performed using a human ocular isolate of *Acanthamoeba castellanii* obtained from the American Type Culture Collection (ATCC 50492). The *Acanthamoeba* strain was maintained in PYG medium. A basal medium (20 g of proteose peptone and 1.0 g of yeast extract in 900 ml of distilled water) and a 2 M glucose stock solution (18 g of glucose and 1.0 g of sodium citrate•2H_2_O in 50 ml of distilled water) were first prepared. Then, PYG medium was prepared by mixing 900 ml of basal medium, 10 ml of 0.4 M MgSO_4_, 8 ml of 0.05 M CaCl_2_, 10 ml of 0.005 M Fe(NH_4_)_2_(SO_4_)_2_•6H_2_O, 10 ml of 0.25 M KH_2_PO_4_ and 50 ml of 2 M glucose stock solution. The *Acanthamoeba* cells were resuspended in fresh PYG medium prior to experiments. *Pseudomonas aeruginosa* (PAO-1) was cultured on heart infusion agar for 24 h at 35 °C, and suspended in PYG medium for use. Based on minimum inhibitory concentration (MIC) test performed by the broth microdilution method as described by the CLSI guideline^17^, *Pseudomonas aeruginosa* PAO-1 was susceptible to 1 μg/ml of levofloxacin (LVFX; Santen Pharmaceutical Co., Japan). *Acanthamoeba* cells were counted in a hemocytometer and adjusted to a density of 1 × 10^5^/ml in PYG medium. *Pseudomonas aeruginosa* was added to the *Acanthamoeba* suspension to obtain a final bacterial density of 3 × 10^5^/ml (low density) or 1 × 10^8^/ml (high density). The two organisms were co-incubated at 25 °C for 2 h before corneal inoculation.

### Animals

We used 24 healthy Japan albino rabbits aged 10–12 weeks and weighing 2.5–3.0 kg. Tokyo Medical University Ethics Committee approved this study involving rabbits. Rabbits used in this study were treated and maintained in rigid accordance to the ARVO Resolution on the Use of Animals in Research. All corneas were examined using a slit lamp before inoculation to exclude any abnormality.

### Inoculation technique

Inoculation of *Acanthamoeba* alone or with *P*. *aeruginosa* was performed as described previously^18^. The right eye of each rabbit was tested and the left eye served as control. Briefly, general anesthesia was induced by intravenous injection of 0.5 ml/kg of 3% sodium pentobarbital, and one drop of benoxinate was applied to the right eye prior to inoculation. Under an operation microscope (Leica-M841, Germany), a 26 G needle attached to a microliter syringe was advanced to the center of the cornea, and 30 μl of a suspension containing 1 × 10^5^ *Acanthamoeba* cells/ml with or without *P*. *aeruginosa* was injected into the corneal stroma.

### Clinical evaluation and end point

The anterior segments of both eyes were examined by a slit-lamp in all rabbits, every day for 5–7 days after inoculation. Inflammatory findings were scored as described previously^19^. The clinical end point was defined as the development of fulminant keratitis in the rabbit cornea, or on day 5 after inoculation when the rabbit cornea remained clear during the study. When the clinical end point was reached, the rabbit was euthanized by an intravenous injection of sodium pentobarbital. The eyes were enucleated for examinations.

### Comparison of infection by *Acanthamoeba* with low-density *P. aeruginosa* treated with 1.5% LVFX eye drop and 1.5% LVFX + BSP eye drops

The effects of topical BSP in rabbit corneas infected by *Acanthamoeba* with a low density (3 × 10^5^/ml) of *P*. *aeruginosa* were examined in 4 eyes of 4 rabbits in each group. Rabbits inoculated with *Acanthamoeba* plus low*-*density *P*. *aeruginosa* were treated topically with 1.5% LVFX eye drops alone or 1.5% LVFX + BSP eye drops starting immediately after inoculation on day 0, 3 times a day until day 5. In the third group, rabbit eyes inoculated with *Acanthamoeba* plus low*-*density *P*. *aeruginosa* were treated immediately with 1.5% LVFX eye drops and then 12 h later with BSP eye drops, 3 times a day until day 5.

Clinical evaluations were performed as described above. Rabbits were euthanized when the clinical end point was reached.

### Comparison of infection by *Acanthamoeba* with high density *P. aeruginosa* treated with 1.5% LVFX eye and 1.5% LVFX + BSP eye drops

The effects of topical BSP eye drops in rabbit corneas infected by *Acanthamoeba* with a high density (1 × 10^8^/ml) of *P*. *aeruginosa* were examined in 6 eyes of 6 rabbits in each group. Rabbits inoculated with *Acanthamoeba* plus high-density *P*. *aeruginosa* were treated with 1.5% LVFX eye drops or 1.5% LVFX + BSP eye drops immediately after inoculation, 3 times a day until day 7. Clinical evaluations were performed as described above. The rabbits were euthanized when the clinical end point was reached.

### Histological evaluation

The corneas of rabbits inoculated with *Acanthamoeba* and high-density *P*. *aeruginosa* treated with 1.5% LVFX + BSP eye drops were examined for the presence of *Acanthamoeba*. The cornea removed from the enucleated eye was mounted in embedding compound (Tissue-Tek OCT, Miles Scientific, Naperville, II) and snap-frozen in liquid nitrogen. Sections were cut at a thickness of 5 μm on a cryostat at −30 °C and were mounted on polylysine-coated slides. All the sections were stained initially by Fungiflora Y and then by PAS. At least ten sections were examined for each eye. Histological localization of *Acanthamoeba* cysts was evaluated.

### Statistical analysis

The data obtained were compiled into a database (Excel Microsoft Windows) and analyzed using statistical software (MedCalc). Mean and standard deviation for each group were calculated for analysis of quantitative variables, and Mann-Whitney tests were used to determine differences among groups. The significance level was set at p < 0.05.

## Supplementary information

Supplemental File 1

## Data Availability

The datasets generated during and/or analyzed during the current study are available from the corresponding author on reasonable request.
