# Supplementary material for: Corticosteroid eye drop instillation aggravates the development of Acanthamoeba keratitis in rabbit corneas inoculated with Acanthamoeba and bacteria
Source: Sci Rep. 2019 Sep 6;9:12821. doi: 10.1038/s41598-019-49128-7 (PMC6731293; doi:10.1038/s41598-019-49128-7)
Supplement: Supplementary file 1 — Supplemental File 1 [file 41598_2019_49128_MOESM1_ESM.pdf]

Supplementary Information

Corticosteroid eye drop instillation aggravates the development of *Acanthamoeba* keratitis in rabbit corneas inoculated with *Acanthamoeba* and bacteria

Hayate Nakagawa, *MD, PhD* [1*], Naohito Koike, *MD, PhD* [2], Tomoko Ehara, *PhD* [2], Takaaki Hattori, *MD, PhD*[1], Akitomo Narimatsu, *MD*[1], Shigeto Kumakura, *MD, PhD* [1], Hiroshi Goto, *MD, PhD* [1]

Supplemental Figure 1

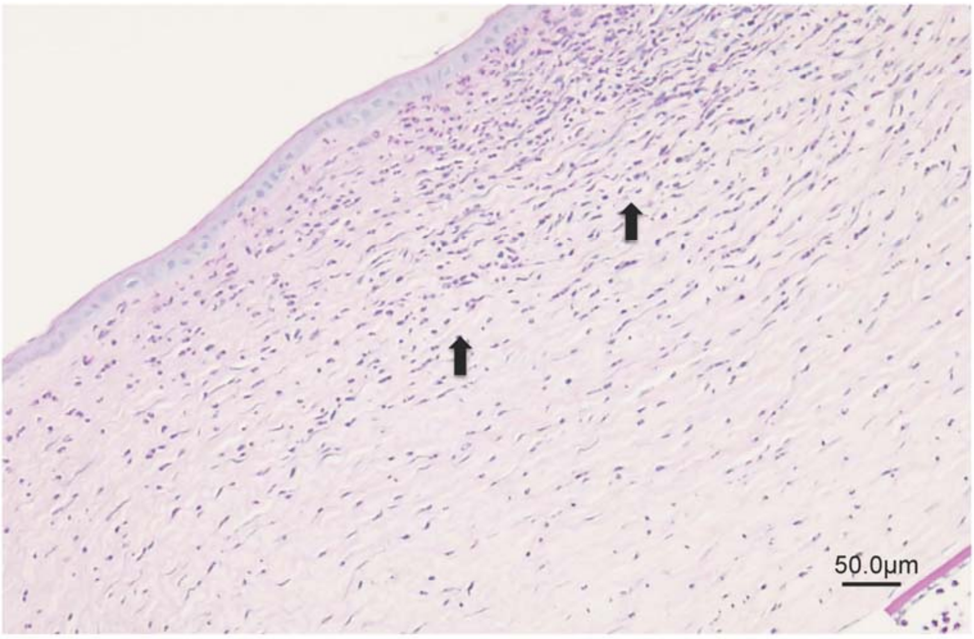

Supplementary Figure 1. Histological finding of corneal tissue inoculated with *Acanthamoeba* plus low-density *P. aeruginosa* and treated with levofloxacin immediately and with BSP 12 hours later. PAS staining. Mild corneal infiltration (↑) is observed.
